# Acceptance and Commitment Therapy for Inpatients with Psychosis (the REACH Study): Protocol for Treatment Development and Pilot Testing

**DOI:** 10.3390/healthcare5020023

**Published:** 2017-05-05

**Authors:** Brandon A. Gaudiano, Carter H. Davis, Gary Epstein-Lubow, Jennifer E. Johnson, Kim T. Mueser, Ivan W. Miller

**Affiliations:** 1 Butler Hospital, Providence, RI 02906, USA; chdavis@butler.org (C.H.D.); gary_epstein-lubow_MD@brown.edu (G.E.-L.); ivan_miller@brown.edu (I.W.M.); 2 Warren Alpert Medical School, Brown University, Providence, RI 02912, USA; 3 College of Human Medicine, Michigan State University, Flint, MI 48502, USA; jennifer.johnson@hc.msu.edu; 4 Center for Psychiatric Rehabilitation, Boston University, Boston, MA 02215, USA; mueser@bu.edu

**Keywords:** hospitalization, psychotic disorders, psychotherapy, pilot projects

## Abstract

Patients with schizophrenia-spectrum disorders frequently require treatment at inpatient hospitals during periods of acute illness for crisis management and stabilization. Acceptance and Commitment Therapy (ACT), a “third wave” cognitive-behavioral intervention that employs innovative mindfulness-based strategies, has shown initial efficacy in randomized controlled trials for improving acute and post-discharge outcomes in patients with psychosis when studied in acute-care psychiatric hospitals in the U.S. However, the intervention has not been widely adopted in its current form because of its use of an individual-only format and delivery by doctoral-level research therapists with extensive prior experience using ACT. The aim of the Researching the Effectiveness of Acceptance-based Coping during Hospitalization (REACH) Study is to adapt a promising acute-care psychosocial treatment for inpatients with psychosis, and to pilot test its effectiveness in a routine inpatient setting. More specifically, we describe our plans to: (a) further develop and refine the treatment and training protocols, (b) conduct an open trial and make further modifications based on the experience gained, and (c) conduct a pilot randomized controlled trial in preparation for a future fully-powered clinical trial testing the effectiveness of ACT.

## 1. Background

Schizophrenia-spectrum disorders (SSD), such as schizophrenia and psychotic mood disorders, are frequently disabling illnesses present in approximately 3.5% of the general population [1]. Schizophrenia is among the top 10 leading causes of disability worldwide [2], and the most disabling psychiatric disorder [3]. In 2002, the total estimated cost of schizophrenia alone in the U.S. was $62.7 billion, with $22.7 billion in direct treatment costs [4]. Patients with SSD represent 25–38% of psychiatric hospital admissions [5,6,7,8]. These patients frequently require hospitalization for safety issues [9,10]. Recurrent inpatient admissions, which are often required during the acute phase of illness for crisis management and stabilization, are key determinants of the high costs of SSD [11]. Reducing relapses and preventing rehospitalizations are important treatment priorities for the management of persons with SSD.

The post-discharge period is a time of increased risk associated with a host of negative outcomes [12,13,14,15]. For example, a Danish study showed that suicide rates were highest within the first 5 days post-discharge and increased with multiple admissions [12]. In addition, research shows that only 50% of patients with SSD achieve functional and symptomatic recovery by 6 months post-hospitalization and many fail to maintain it [16]. In another study of 76 hospitalized patients with schizophrenia, 38% were rehospitalized at least once over 18 months [17]. Frequent hospitalizations cause significant disruptions to patients’ lives. Also, inpatients are at heightened risk for treatment nonadherence following discharge [18] and nonadherence is predicted by inadequate discharge preparation [19,20]. Thus, patients with SSD are at high risk for subsequent relapse and rehospitalization following an acute hospitalization.

Cognitive-behavioral therapies (CBT) for psychosis are efficacious based on data from randomized controlled trials conducted mainly in outpatient samples showing superiority over treatment as usual (TAU) alone, which typically includes antipsychotic pharmacotherapy [21,22,23,24,25]. However, few trials of traditional CBT for psychosis have been conducted in inpatient samples [21]. All such trials thus far have been conducted in Europe and provided during longer-term inpatient care [26,27] and/or started during hospitalization and continued through outpatient care [28]. Comprehensive CBT, particularly in hospital settings, is rarely provided to patients with schizophrenia in the U.S. [29]. One implementation issue is that short acute hospital stays (e.g., 1 week or less) require brief interventions and little previous research has systematically addressed this issue [9,30]. As medications are the primary strategy used for stabilization, there is a lack of qualified therapists on inpatient units who can deliver evidence-based psychosocial interventions [31]. Nonetheless, there is a high degree of interest among inpatient staff and patients for increased availability of psychosocial interventions during hospitalization [31,32].

Recently, psychosis researchers have begun investigating innovative new forms of CBT that emphasize acceptance (i.e., willingness to experience unavoidable psychological distress) and mindfulness (i.e., nonjudgmental, present-moment attention) strategies [24,33]. The only form of CBT for psychosis that has been tested in randomized controlled trials in short-term hospital settings in the U.S. to date is called Acceptance and Commitment Therapy (ACT). ACT is a newer form of CBT that emphasizes acceptance and mindfulness instead of challenging the content of dysfunctional thoughts (i.e., cognitive restructuring), and focuses on behavior change strategies guided by the patient’s personal values to improve functioning and coping with persistent symptoms [34]. ACT is currently listed as an empirically-supported psychotherapy by the American Psychological Association [35] for a number of psychiatric conditions, including psychosis, and is further recognized by the U.S. Substance Abuse and Mental Health Services Administration [36] and U.S. Veterans Administration [37]. Recent meta-analyses demonstrate that ACT is at least as effective as more traditional forms of CBT and is efficacious in terms of both acute and longer-term effects on symptoms and functioning for a wide variety of conditions, including psychosis, borderline personality disorder, chronic health conditions (e.g., pain, epilepsy, diabetes), substance abuse, depression, and anxiety disorders [38,39,40]. Finally, a recent meta-analysis focused specifically on early mindfulness and acceptance therapies for psychosis, including ACT, showed medium effect size improvements on positive and negative symptom outcomes through follow-up compared to comparison conditions, and changes in mindfulness and acceptance predicted changes in symptoms [41].

As mentioned, previous research shows that ACT is feasible and efficacious for treating psychosis specifically in acute-care hospital settings. In the first randomized controlled trial conducted in a mixed sample of 80 inpatients with SSD, Bach and Hayes [42] found that a brief, 4-session, individual ACT intervention reduced the believability of psychotic symptoms and rates of rehospitalization (20% vs. 40%) over 4 months post-discharge compared to a TAU condition. The advantage for ACT over TAU in terms of rehospitalization rate was maintained at 1 year follow-up [43].

Gaudiano and Herbert [44] conducted a study that improved on Bach and Hayes’ methodology by adding an established measure of symptom severity (the Brief Psychiatric Rating Scale [45]) and using an enhanced TAU condition that controlled for therapist contact time. Among a sample of 40 patients with SSD, they found that an average of 3 individual therapy sessions of ACT over 1 week of inpatient hospitalization resulted in greater improvement in clinically significant symptom changes (especially mood symptoms), disability related to illness, and distress related to psychotic symptoms by hospital discharge. A survival analysis controlling for baseline symptom severity showed that ACT produced a significantly longer time to rehospitalization at 4 month follow-up compared to TAU [46]. Furthermore, evidence was found to support the proposed mechanisms of action of ACT for psychosis. Changes in believability of psychotic symptoms mediated the effects of treatment condition on levels of distress about psychotic symptoms at hospital discharge [47]. In addition, changes in believability of psychotic symptoms mediated the effects of treatment condition on 4-month rehospitalization rates in an analysis of the combined datasets from the Bach and Hayes and Gaudiano and Herbert studies [46].

Thus, ACT is the only specific psychosocial treatment found to be feasible and efficacious for inpatients with psychosis with shorter hospital stays in the U.S. based on randomized clinical trials to date. However, both the Bach and Hayes [42] and Gaudiano and Herbert [44] studies provided brief treatment, lacked blind raters, and assessed a limited range of outcomes, especially post-hospitalization. In addition, these studies were conducted by ACT-trained research therapists using an individual-only therapy format without integration into the hospital unit milieu (i.e., group therapy and other interactions with unit staff), limiting the potential implementability of the intervention in routine inpatient settings. Not surprisingly, then, the uptake of ACT on inpatient units has been slow and inconsistent to date.

### 1.1. Summary

Inpatients with SSD face a number of challenges, including inadequate functional improvement from current treatments, a high risk of relapse/rehospitalization, increased suicide risk, and nonadherence to post-discharge treatment plans. Even though a growing body of research conducted mainly in outpatient samples shows that psychosocial treatments for psychosis can significantly improve outcomes compared to TAU alone, these interventions have not been systemically adapted and tested in U.S. hospitals. ACT is an empirically-supported therapy with efficacy for treating a range of conditions. Importantly, ACT is the only psychosocial treatment demonstrating efficacy for treating inpatients with SSD during short hospital stays in the U.S. ACT teaches patients skills to cope with persistent psychotic symptoms using innovative mindfulness strategies that can be helpful for targeting functional impairment, reducing relapses and thus rehospitalizations and emergency visits, decreasing symptoms commonly associated with psychosis (e.g., depression), and improving treatment engagement. However, the previously-tested ACT research intervention will require further adaptation and refinement to promote implementation in typical hospital settings.

### 1.2. Rationale for the Current Project

This paper describes our initial plan for the Researching the Effectiveness of Acceptance-based Coping during Hospitalization (REACH) Study, which is currently underway. The goal of REACH is to develop a model of treatment that facilitates the integrated delivery of psychosocial treatments in the inpatient setting by adapting ACT to: (1) use a multi-modal format (individual and group sessions), (2) be delivered by inpatient clinicians typically working in these settings (e.g., social workers, nurses, occupational therapists), (3) and be designed for patients with a spectrum of psychotic disorders as part of their routine acute hospital care. We propose to test the intervention’s effectiveness compared with routine hospital care in a future large-scale randomized controlled trial. In the longer term, we expect ACT for Inpatients (ACT-IN) to improve acute (e.g., overall psychiatric symptoms and associated distress) and post-hospital outcomes (i.e., rehospitalization, psychosocial functioning). We also expect ACT-IN to be acceptable to patients and hospital stakeholders, and ultimately implementable in a typical inpatient setting. The first step in this process is to refine and test the ACT-IN intervention and develop appropriate research procedures and training methods by conducting initial open and pilot randomized trials of the newly adapted ACT-IN intervention in the current project.

## 2. Method

### 2.1. Study Design

**Figure 1:**
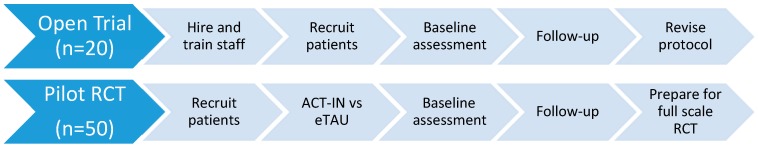
Study design.

First, an Open Trial Phase will be conducted to further refine and adapt study protocols in preparation for the subsequent Pilot Randomized Controlled Trial (RCT) Phase (see Figure 1). The open trial (*n* = 20) will be conducted over the first year of the project and the pilot RCT (*n* = 50) will be conducted during years two and three. Recruitment and refinement of the ACT-IN intervention, fidelity scale, and study procedures will occur in an iterative process of review based on feedback from patients/therapists and examination of data collected. Following the conduct of the open trial and refinement of the protocol based on feedback and experience gained during this process, the pilot RCT will be conducted. In addition to typical hospital treatment (i.e., pharmacotherapy), patients will be randomized to receive either ACT-IN or enhanced TAU (eTAU) during their hospital stay that controls for contact time. After completion of the open and pilot randomized trials, the treatment protocols and procedures will be refined in preparation for a future full-scale clinical trial of ACT-IN.

### 2.2. Sample

Participants will be inpatients recruited from Butler Hospital, a 180-bed private, non-profit psychiatric and substance abuse hospital located in Providence, RI. Inclusion criteria include: (1) current psychiatric hospitalization; (2) DSM-5 diagnosis of a primary psychotic disorder or mood disorder with psychotic features (schizophrenia, schizoaffective disorder, schizophreniform, delusional disorder, psychotic disorder unspecified, bipolar disorder with psychotic features, major depressive disorder with psychotic features) as determined by structured clinical interview [48]; (3) 18 years or older; and (4) ability to speak and read English. Exclusion criteria include: (1) psychosis severe enough to prevent participation in regular hospital therapy groups; (2) psychosis related to a general medical condition or substance-induced psychotic disorder; or (3) severe cognitive impairment (Mini-Mental State Exam < 15) [49].

We chose to include patients with schizophrenia-spectrum disorders, including psychotic mood disorders, as the treatment is designed to be applicable to patients with a wide range of symptoms. Patients will likely display a range of severity and treatment will be tailored to higher and lower functioning patients. Based on our pilot work [44], patients judged by their psychiatrist to be stable enough to participate in routine group therapy will be eligible to participate in the study. Typically, patients are encouraged to begin attending hospital groups when they are stabilized enough on medications to remain in the group setting without disruption (within first 1–2 days after admission). Patients will not be excluded due to negative symptoms (e.g., flat affect), but will be excluded if determined to exhibit severe cognitive impairment due to potential difficulty participating in research. Mild to moderate cognitive impairment will be permitted.

### 2.3. Recruitment Procedures

Ethical approval for conducting the study is approved through the Butler Hospital IRB. If the patient is appropriate based on initial chart review of hospital admissions (and after obtaining a HIPPA waiver for this purpose), the study will be explained to the patient’s psychiatrist. If the psychiatrist agrees, a member of the research team will then approach the patient. The nature, purpose, and risks and benefits of the study will be explained to the patient and informed consent will be obtained. All research staff will be trained in ethical research procedures with severely mentally ill patients.

### 2.4. Randomization

Patients will be assigned to conditions in the pilot RCT in a 1:1 ratio, with 2 stratification variables: gender and diagnosis (primary psychotic disorder vs. psychotic mood disorder). For example, gender is a theoretically important stratification variable because women with schizophrenia tend to have better premorbid functioning, a later age of onset, a different symptom profile, and a better course of illness compared with male patients [50]. Randomization will be conducted by a computer program with no research staff aware of assignments in advance.

### 2.5. Treatment Conditions

#### 2.5.1. Enhanced Treatment As Usual (eTAU)

According to National Institute of Mental Health’s *Road Ahead* [51] report: “policy makers need to know if a new program works better or costs less with similar effectiveness than what is currently available, or if it is better than doing nothing at all” (p. 10). For a variety of ethical, pragmatic, and research concerns, the most commonly used comparison group in severe mental illness research has been some variant of “treatment as usual” (TAU) [21]. Although use of TAU as a comparison group has some limitations, it also has advantages when applied to severe and chronic mental illness in which patients typically participate in multiple treatments. Since ACT-IN is designed to be an “adjunctive” intervention to be delivered as part of routine hospital care alongside other treatments, the use of TAU seems particularly appropriate.

The TAU for all patients regardless of condition will consist of hospital treatment, including daily medication management sessions with a psychiatrist (e.g., antipsychotic and other medications as appropriate), community meetings, other group therapy, case management, occupational therapy, open air walks, discharge planning, physical examination, and other services (e.g., medical testing). Discharge plans include an appointment with a medication provider scheduled shortly after discharge. Patients receive referrals/appointments for other follow-up treatment as needed (e.g., individual therapy).

TAU will be “enhanced” (eTAU) in the following ways to better account for treatment format and contact time. Patients in the pilot RCT assigned to eTAU will receive a similar number of individual and group sessions as provided to ACT-IN patients to account for contact time (see below). Previous research has found that “nonspecific”, supportive interventions are often efficacious when compared to other specific interventions for psychosis [52]. However, given the effectiveness aims of our project, we believed that it would be most appropriate to compare our ACT intervention with the types of treatment typically delivered on inpatient units. Thus, eTAU will involve the provision of routine hospital treatment that does not contain ACT-IN content. eTAU group content includes: (1) symptoms group (i.e., psychoeducation about illness is provided and patients share their experiences about what is helpful for coping with symptoms); (2) general cognitive-behavioral skills (i.e., discussion of coping with negative cognitions and engaging in pleasant activities); (3) medications group (i.e., including discussion of their purposes and side effects and how to manage daily medications); and (4) relaxation group (i.e., activities that can aid in relaxation). Furthermore, individual eTAU sessions will focus on providing general supportive therapy as adapted from previous protocols [53]. We considered providing patients with a specific psychosis comparison intervention (e.g., traditional CBT for psychosis), but decided against this given the effectiveness-implementation aims of the project. It is typical for patients to receive a combination of group and individual sessions during their hospital stay, just not focused on the ACT model.

#### 2.5.2. Acceptance and Commitment Therapy (ACT)

Social, cultural, and developmental contexts are important for understanding the impairment and dysfunction that result from psychotic symptoms [54]. Consistent with this perspective, ACT belongs to a family of cognitive-behavioral treatments philosophically rooted in functional contextualism [34]. A functional contextual approach “views psychological events as ongoing actions of the whole organism interacting in and with historically and situationally defined contexts” [38] (p. 4). The goal of functional contextualism is to achieve personal “workability” in a given context. Thus, newer functional models of psychopathology including ACT emphasize that impairment is influenced by the patient’s response to and interaction with their symptoms, and not only the presence of the symptoms themselves [55,56,57,58].

We believe that ACT holds advantages over other models because its contextual approach is particularly consistent with a recovery-oriented model [59] as it focuses on commitment toward personal values and goals, as well as improvement in functioning and quality of life rather than symptoms alone. In ACT, treatment is guided by the individual’s chosen, personal life values and patients are encouraged to advocate for their desires and needs to build resiliency to uncontrollable life events. Also, techniques are chosen in ACT that are most personally relevant and culturally appropriate for the individual. We believe such features are essential to the development of a consumer-driven approach to inpatient psychosocial treatment.

In general, the ACT model seeks to target experiential avoidance. Experiential avoidance is proposed to involve the excessive negative evaluation of thoughts and emotions, an unwillingness to experience them, and the resulting efforts made to control or escape them that can interfere with functioning [34,38,58]. For example, a person may be distressed by feelings of anxiety triggered by stressful situations and attempt to control this anxiety by avoiding any triggers, leading to functional impairment. Studies in various clinical samples demonstrate that experiential avoidance is strongly correlated with psychopathology [60,61,62]. Recent work on psychosis has shown that experiential avoidance focused on hallucinations [63], paranoia [64], and delusions [65] all appear to have toxic psychological effects, and contribute to cognitive impairments [66] in schizophrenia. Experiential avoidance is thought to lead to a kind of mental rigidity or inflexibility, which has been observed in hospitalized patients [67,68,69] and those with psychosis [70], which inhibit effective coping efforts. Research demonstrates that chronic experiential avoidance produces a paradoxical effect in that attempts to escape or control unwanted private experiences may actually increase their frequency or intensity over time [71,72,73,74,75,76]. By decreasing experiential avoidance though various strategies described below, ACT reduces the intensity of and distress related to psychosis.

**Figure 2:**
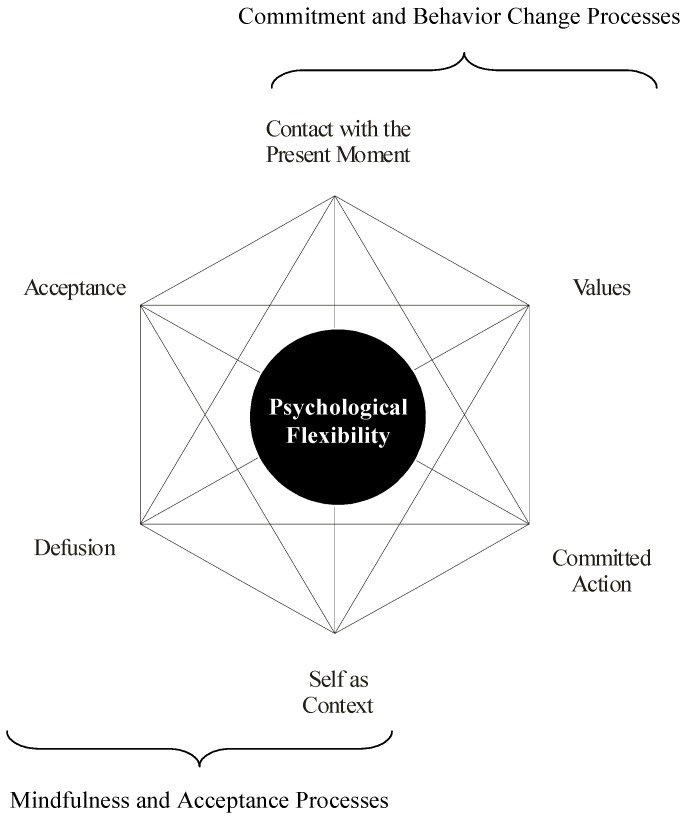
The Acceptance and Commitment Therapy (ACT) model.

Psychological flexibility is the converse of experiential avoidance and the ultimate goal of ACT. Psychological flexibility is defined as being fully present in the moment, and changing or persisting in one’s behavior based on what works best in the service of a person’s chosen values [34]. There are six core intervention strategies used in ACT to increase psychological flexibility briefly summarized below:

- **Acceptance** is defined as one’s willingness to experience unwanted thoughts and feelings in the pursuit of a valued goal.
- **Cognitive defusion** involves taking a meta-cognitive perspective toward cognitions by treating thoughts as thoughts instead of as their literal content. For example, cognitive defusion of paranoid ideation would involve the patient taking the following perspective: “I’m noticing that I’m having the thought right now that…someone is trying to harm me.”
- **Nonjudgmental attention to the present moment** invokes the process of being mindful and aware of one’s ongoing experiences.
- **Flexible perspective-taking** involves recognizing the part of one’s self that is stable, consistent, and independent from transient mental events like thoughts and feelings.
- **Personal values** are defined as global, desired, and chosen life directions that make life worth living.
- Finally, **commitment** in ACT means engaging in values-consistent behavior change efforts (see Figure 2) [38].

Thus, the emphasis in ACT is on changing one’s relationship to symptoms rather than altering their appearance or frequency directly. To achieve these aims, ACT employs a variety of strategies, including vivid metaphors/stories to communicate key treatment concepts and engaging exercises/activities to increase willingness and practice working successfully with the internal distress that accompanies behavior change. These strategies make the treatment more understandable and memorable for patients with psychosis, and help them cope with any persistent symptoms that may otherwise impede their pursuit of valued goals.

### 2.6. ACT for Inpatients (ACT-IN) Protocol

**Figure 3:**
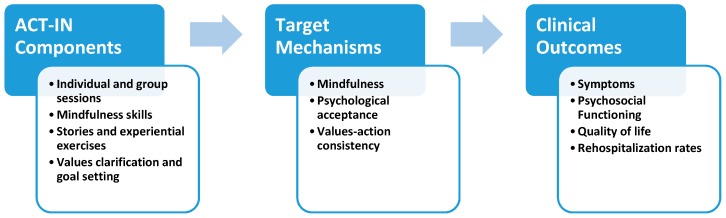
ACT for Inpatients (ACT-IN) components, targets, and outcomes.

The treatment protocols used in the Gaudiano and Herbert [44] and Bach and Hayes [42] studies were adapted for the current study. ACT-IN has multiple components that are theorized to target the mechanisms of mindfulness, acceptance, and values in order to improve symptoms, functioning, and rehospitalization rates following discharge (see Figure 3). Average length of hospital stay for patients with SSD at our recruitment site is about one week. The number of ACT-IN sessions varies based on the length of hospital stay as done in our pilot work [44]. The intervention uses a rolling/open format so that patients can start and stop sessions as their length of stay dictates, with a target of three individual and three group sessions per week. In each session, various mindfulness and acceptance exercises as described by Hayes et al. [34] are introduced to help patients decrease avoidance or struggle with internal experiences, including psychotic symptoms, which cause functional impairment.

We made several modifications to ACT-IN to improve its potentially implementability and effectiveness in routine hospital settings. For example, we included both individual and group sessions to provide a greater “dose” of ACT compared with previous studies that were individual only. This also allows greater consistency of treatment during hospitalization and acknowledges the fact that group therapy is a major component of psychosocial treatment on most inpatient units. We considered administering ACT-IN in a group format alone to maximize the potential for easy uptake in routine hospital settings. However, we concluded that less experienced clinicians may not be able to provide a sufficient amount of treatment to achieve optimal clinical improvement. The combination of individual and group sessions balances these concerns and recognizes that both formats are used to provide brief psychosocial intervention on many units today. In addition, we developed training procedures and a treatment protocol designed for providers who may not have had previous experience with ACT or other evidence-based psychosocial approaches for psychosis. The ACT-IN treatment manual is presented in a simplified format with more structure and examples than other manuals to facilitate delivery by less experienced clinicians. Group sessions are designed to be conducted in an open format so that patients can participate in sessions as their length of stay dictates. In addition, the treatment is designed to be applicable to patients with a wider range of diagnoses (e.g., primary psychotic and psychotic mood disorders) and presentations (e.g., those with lower cognitive functioning and comorbid conditions) given the higher levels of complexity and acuity found in patients treated on inpatient units (as described below).

The final content of ACT-IN will be developed based on the lessons learned during the current project. However, what follows represents our initial plans for the intervention. The first ACT-IN individual session with the patient is focused on introducing the ACT model, establishing rapport, collecting history of illness to highlight the pernicious role that avoidance plays in exacerbating symptoms, and identifying values-consistent behavioral goals that the patient will work on during treatment. Subsequent individual and group sessions adhere to the following general format.

First, the ACT model is explained. Then, the therapist guides patients through an exploration of previous attempts to cope with symptoms, the successfulness or unsuccessfulness of these strategies, and their consequences. Unhelpful attempts to avoid or struggle with these symptoms are explored in the context of their “workability” to achieve a valued goal. The therapist highlights attempts by patients to avoid or struggle with symptoms that paradoxically seem to cause increased distress, intensity, or impairment. The therapist then notes that it is important not just to consider the effects of the symptoms themselves, but also how one responds to them.

Next, patients are taught various mindfulness and acceptance strategies to nonjudgmentally observe and cognitively defuse from their thoughts, and to notice that they can have symptoms without acting on them or trying to change them. Intensive meditation practices are generally contraindicated for patients with acute psychosis [77]. Mindfulness exercises are modified to be appropriate for this patient population by making them “eyes open”, briefer in duration, and focused on bringing nonjudgmental awareness to external stimuli in the present moment (e.g., eating, walking). For example, the therapist leads the patient/group in a brief (5–10 min) mindfulness exercise in which individuals are instructed to eat a piece of food (e.g., a raisin) or to walk around quietly in a mindful fashion while staying present. During this mindfulness activity, patients are instructed to notice their various sensory experiences one at a time (e.g., noticing how their foot feels when contacting the ground repeatedly).

Furthermore, the concept of values is introduced and explored during sessions. Values are described as the things that give a person’s life meaning and purpose and are individually chosen. The therapist clarifies possible values that may be relevant to each patient, including family, romantic relationships, work, leisure, and health. The values clarification discussion involves developing a more in-depth description and elaboration of valued areas based on a patient’s personal life situations. Discrepancies between the person’s valued area and his/her daily actions are highlighted along with the distress this tends to cause. The therapist suggests that being more consistent with values in one’s daily actions can lead to a more fulfilling and healthy life. Specific behavioral goals are then discussed and a written plan for implementing them after the session is developed in collaboration with the patient.

Sessions end with a discussion of “take home” messages and ways of implementing the strategies learned in between sessions. Following the same basic format as described above, different ACT “themes” are rotated across sessions and include: values clarification, mindfulness, acceptance, and cognitive defusion. In each session, various stories/metaphors and experiential exercises are implemented relevant to each ACT theme. Patients are encouraged to practice exercises and to work toward valued goals between sessions. For an extended discussion of ACT for psychosis strategies see Gaudiano (2013) [78].

### 2.7. Study Therapists

We will use staff typically employed on hospital units as study therapists, including social workers, master’s level counselors, nurses, and occupational therapists. Therapists will be trained to: (a) understand and learn to perform the treatment protocols consistently across patients and (b) avoid using techniques that are not part of the treatment. Educational materials are drawn from the treatment manual on the conceptualization, components, and delivery of the intervention. The teaching materials include video role plays to demonstrate appropriate vs. incorrect interventions, plus strategies to manage patients who may have difficulty using techniques. A didactic training workshop is used, in which instructors present the material, answer questions, and role-play various strategies and situations. Following the didactic training, study therapists participate in “mock” sessions in which they deliver intervention content with study staff acting as confederates. These sessions are recorded and assessed for fidelity and competency. Ongoing supervision will be provided to therapists throughout the study to problem solve issues and to prevent protocol “drift”. Remedial training and practice will be provided to therapists who do not maintain acceptable fidelity.

### 2.8. Treatment Fidelity

An initial version of the ACT-IN fidelity scale was developed to assess therapists’ adherence to the protocol and skill in delivering each treatment component in group and individual formats, as well as in developing a positive therapeutic environment (i.e., empathy, warmth, flexibility). The goal was to create a usable scale that could be employed in teaching ACT to staff, as well as for evaluating fidelity to ACT. Items were generated based on the treatment manuals and existing fidelity scales used in previous studies [79,80]. Sessions will be recorded and a random selection of sessions will be coded for fidelity by research staff trained to initial interrater reliability.

### 2.9. Assessments

Assessments will be conducted shortly after hospital admission (baseline), at hospital discharge (or shortly thereafter for unexpected discharges), and 4 months post-discharge (as done in the initial ACT inpatient studies). At admission, clinical interviews will assess the week immediately preceding hospitalization, the discharge assessment will assess current severity/functioning (past 24 h), and the follow-up assessment will assess the previous week. We will assess diagnosis, psychiatric symptom severity including psychosis, psychosocial functioning and quality of life, treatment adherence and utilization (including rehospitalization rates at follow-up), treatment satisfaction and acceptability, and the proposed mechanisms of ACT-IN (mindfulness, acceptance, values). Severity of positive and negative symptoms will be assessed using the relevant subscales from the BPRS, which have demonstrated reliability and validity for this purpose [81]. A list of measures with a brief description of each is provided in Table 1.

### 2.10. Assessors

Assessors will be blind to treatment assignment. All assessors will receive comprehensive training including: (a) review of assessment manuals and training recordings, (b) supervised role-playing, and (c) practice interviews. We will follow training procedures for the BPRS developed by Ventura et al. [82] involving demonstration of reliability via ratings of 6 standardized clinical interview recordings. Prior to administering study assessments, all assessors will be required to achieve adequate reliability (ICC ≥ 0.80) with other trained raters, and then undergo periodic checks to prevent “drift”. During the study, all interviews will be recorded and periodically checked for accuracy. Should any interviewer inaccuracy or drift be detected, further training will be provided.

### 2.11. Retention Efforts

Our goal will be to achieve <20% attrition over the course of the study. However, we will assess whether attrition is higher than expected and use this information to determine feasibility and sample size needed for a future large-scale clinical trial. Subjects will be compensated for completing assessments ($25 each for the admission and discharge assessments, and $50 for the follow-up assessment). Assessors will phone patients periodically during follow-up to maintain contact and update information. Cab rides/bus passes will be provided for subjects unable to transport themselves to our center for their follow-ups. We will attempt to continue to collect assessment data even if patients withdraw from the study.

## 3. Sample Size and Data Analysis Considerations

As recommended for pilot projects [92], we did not power the study for effect sizes at certain *p* values due to the large confidence intervals around effects in smaller samples. Instead, the primary objective of the REACH treatment development project is to establish acceptability/feasibility and specify study procedures in preparation for a *future* fully-powered clinical trial [92]. However, a preliminary power analysis is provided below as a guide for a future large-scale clinical trial should the pilot project prove successful. In order to have an 80% power to detect group differences at a two-tailed alpha level of .05 over 3 time points, a small treatment effect (f = 0.10) would require a total *n* = 526, a medium effect (f = 0.25) would require *n* = 86, and a large effect (f = 0.40) would require only n = 36. Clinically meaningful effect sizes on symptom outcomes (BPRS) are expected to be medium to large in magnitude and should place future sample size requirements in a feasible range. We should be able to gauge relevant treatment parameters with an open trial sample of 20 and a pilot RCT sample of 50. This will require recruitment of approximately 3–4 patients per month into study, which is feasible based on the admission rate at our study site and the recruitment rate achieved by the investigators in their previous studies.

We will examine effect size standard errors, confidence intervals, and clinically meaningful statistics (e.g., “number needed to treat”) as recommended for treatment development trials [93]. Furthermore, we will examine patterns of missing data and explore multiple methods for imputing data to determine consistency of results (maximum likelihood estimation, multiple imputation, and “worst case” scenario). If baseline differences are identified and associated with outcomes, these variables will be used as covariates in subsequent analyses. For example, history of illness (e.g., first episode versus chronic psychosis) may be important to examine due to its relationship with outcome in schizophrenia [94].

Regarding treatment feasibility/acceptability, we will assess attrition rates and reasons for drop out. We also will assess levels of treatment acceptance and satisfaction (CSQ-8). Similar to other studies in this area [95,96], the primary outcome will be change on the BPRS total scale. Secondary outcomes will include symptom subgroups (e.g., positive, negative, mood), overall response rates (≥30% reduction [97] on BPRS), psychosocial functioning and quality of life, and rehospitalization rates. We also will examine potential predictors of outcomes to identify moderators and mediators that could be tested in subsequent research, including demographics, treatment adherence, and the proposed ACT-IN treatment mechanisms (AAQ-II, CAMS-R, VQ).

Given all patients will be receiving appropriate pharmacotherapy as part of their hospital stay, we expect that both groups will show significant improvement in symptom severity by discharge. However, we also are interested in examining whether ACT provides certain advantages in terms of its impact on nonpsychotic symptoms (e.g., mood symptoms) or other outcomes (e.g., psychosocial functioning) compared with eTAU. Consistent with the analyses we would plan to conduct in a future full-scale RCT, we will generate multilevel linear models in a preliminary fashion to examine whether treatment condition, time, and the treatment condition X time interaction predicts change in outcomes (symptoms, functioning, rehospitalization) and proposed mechanisms [98]. These exploratory analyses will follow the intent-to-treat principle (i.e., all patients randomized to treatment). Differences in median days to hospitalization between conditions, log-rank test, and preliminary Cox proportional hazards models will be used to understand treatment effects on time to re-hospitalization during follow-up. In addition, the potential magnitude of the indirect effect of condition on outcomes will be explored in a preliminary fashion by testing between-condition differences in mechanisms targeted by the intervention (path ‘a’) (e.g., mindfulness) and determining whether group differences (path ‘b’) are related to outcomes. The product of path coefficients will provide an estimate of the mediation effect and confidence intervals [99]. We acknowledge we will be underpowered for formal mediation testing. However, the partitioning of variance should provide interpretive guidance regarding the effects of ACT-IN mechanisms when assessing symptomatic outcomes.

## 4. Discussion

The REACH Study open trial phase was recently completed and data are being analyzed. Recruitment for the pilot RCT is currently underway. We anticipate that further modifications will be made to the ACT-IN protocol and study procedures based on “lessons learned” during the conduct of the project. These results and changes will be presented in subsequent publications stemming from the project. Upon final completion of the REACH treatment development project, a series of meetings will be held with the investigators to review: (1) qualitative exit interviews from patients; (2) treatment protocols in light of outcomes achieved; (3) therapists’ fidelity and satisfaction; (4) assessor training procedures; (5) recruitment/retention rates (especially with ethnic/minority participants); and (6) other stakeholder feedback (e.g., administrators). We also will use this time to analyze data and prepare manuscripts. These final revisions of protocols, manuals, and scales will represent the final “products” of the project and will be used in a future test of the effectiveness of ACT-IN in a fully-powered clinical trial.

## 5. Conclusions

Acute hospitalization provides a unique opportunity to teach patients new skills and coping strategies to influence the course of illness in schizophrenia-spectrum disorders. However, there has been a paucity of research on the development, testing, and dissemination of adjunctive psychosocial interventions for psychosis in routine hospital settings in the U.S. The REACH Study will lay the groundwork for a future, fully-powered clinical trial evaluating the effectiveness of a novel psychosocial intervention for improving outcomes in high-risk, hospitalized patients with psychosis. If successful, the current project will address the important problem of hospitalization in patients with psychosis, and inform the development of psychosocial treatments that can address the longer-term implications for improving patients’ functioning post-discharge and supporting recovery.

## Figures and Tables

**Table 1:** ACT-IN components, targets, and outcomes.

Measure	Construct Assessed	Method	Time Points
**Screening and Diagnosis**			
Mini-Mental state Exam (MMSE) [49]	Cognitive impairment	Interviewer	BL
Structured Clinical Interview for DSM-IV (SCID) [48]	Axis I diagnosis	Interviewer	BL
**Psychiatric Symptoms**			
Brief Psychiatric Rating Scale (BPRS) [45,82]	Psychiatric symptoms	Interviewer	BL,DC,4
Clinical Outcomes in Routine Evaluation (CORE) [83]	Psychiatric symptoms	Self-Report	BL,DC,4
**Functioning**			
Heinrichs Brief Quality of Life Scale [84]	Quality of Life	Interviewer	BL,DC,4
WHO Disability Assessment Schedule (WHODAS)-12 [85]	Psychosocial Functioning	Self-Report	BL,DC,4
Schizophrenia-Quality of Life-18 (S-QOL-18) [86]	Quality of Life	Self-Report	BL,DC,4
**Treatment Adherence/Utilization**			
Brief Adherence Rating Scale (BARS) [87]	Medication adherence	Interviewer	4
Treatment History Interview-4 (THI-4) [88]	Treatment utilization	Interviewer	4
**Mechanisms Measures**			
Cognitive and Affective Mindfulness Scale-Revised	Mindfulness	Self-report	BL,DC,4
Acceptance and Action Questionnaire-II (AAQ-II) [61,89]	Psychological acceptance	Self-report	BL,DC,4
Valuing Questionnaire (VQ) [90]	Values-consistent living	Self-report	BL,DC,4
**Acceptability/Satisfaction**			
Client Satisfaction Questionnaire-8 (CSQ-8) [91]	Treatment satisfaction	Self-report	DC
Qualitative Post-Treatment Interview	Treatment satisfaction	Interview	DC

Note: BL = Baseline, DC = Discharge, 4 = 4 Month Follow-up.
